# Elevated CRP levels predict poor outcome and tumor recurrence in patients with thymic epithelial tumors: A pro- and retrospective analysis

**DOI:** 10.18632/oncotarget.17478

**Published:** 2017-04-27

**Authors:** Stefan Janik, Christine Bekos, Philipp Hacker, Thomas Raunegger, Bahil Ghanim, Elisa Einwallner, Lucian Beer, Walter Klepetko, Leonhard Müllauer, Hendrik J. Ankersmit, Bernhard Moser

**Affiliations:** ^1^ Department of Thoracic Surgery, Division of Surgery, Medical University of Vienna, Vienna, Austria; ^2^ Christian Doppler Laboratory for Diagnosis and Regeneration of Cardiac and Thoracic Diseases, Medical University of Vienna, Vienna, Austria; ^3^ Department of Laboratory Medicine, Medical University of Vienna, Vienna, Austria; ^4^ Department of Biomedical Imaging and Image-Guided Therapy, Medical University of Vienna, Vienna, Austria; ^5^ Clinical Institute of Pathology, Medical University of Vienna, Vienna, Austria

**Keywords:** thymic epithelial tumors, thymoma, thymic carcinoma, CRP, prognosis

## Abstract

**Objective:**

Scarce information exists on the pathogenesis of thymic epithelial tumors (TETs), comprising thymomas, thymic carcinomas (TCs) and neuroendocrine tumors. C-reactive protein (CRP) increases during certain malignancies. We aimed to investigate the clinical relevance of CRP in patients with TETs.

**Results:**

Pretreatment CRP serum concentrations were significantly elevated in patients with TETs, particularly TCs and metastatic TETs. After complete tumor resection CRP serum concentrations were decreased (*p* = 0.135) but increased significantly in case of tumor recurrence (*p* = 0.001). High pretreatment CRP was associated with significantly worse 5- and 10-year freedom-from recurrence (FFR) (*p* = 0.010) and was a negative prognostic factor for FFR (HR 3.30; *p* = 0.015). IL-6 (not IL-1β) serum concentrations were significantly elevated in patients with TETs but we did not detect CRP tissue expression in TETs.

**Materials and Methods:**

Pretreatment CRP serum concentrations were retrospectively analyzed from 128 surgical patients (1990–2015). In a subset of 68 patients longitudinal analysis of CRP was performed. Additionally, immunohistochemical tumor CRP expression and serum concentrations of interleukin (IL)-6 and IL-1β were measured.

**Conclusions:**

Hence, diagnostic measurement of serum CRP might be useful to indicate highly aggressive TETs and to make doctors consider tumor recurrences during oncological follow-up.

## INTRODUCTION

Thymic epithelial tumors (TETs) are rare malignancies that originate from thymic epithelial cells. They represent the most common tumors of the anterior mediastinum in adults. TETs are differentiated into thymomas, thymic carcinomas (TCs) and thymic neuroendocrine tumors (TNETs) based on fundamental histologic and molecular patterns [1]. According to WHO classification thymomas are classified into type A, AB, B1, B2 and B3 and further rare subtypes [1]. TETs are clinically staged according to the Masaoka-Koga staging system based on their level of invasiveness [2]. TCs usually present with more aggressive tumor behavior, advanced tumor stages and consequently with worse overall survival (OS) and freedom from recurrence (FFR) compared to thymomas [3–5]. TNETs comprise less than 5% of all TETs and are characterized by high biologic aggressiveness and poor prognosis due to high rates of recurrences and tumor related deaths [6, 7]. The occurrence of thymomas is associated with paraneoplastic autoimmune Myasthenia Gravis (MG) in about 30% of patients [8].

Tumor recurrence rates of 17%, 28% and 38% for patients with thymomas, TCs and TNETs were described, respectively [4, 7, 9] Surgical resection of recurrences was proposed as the mainstay of treatment and was associated with prolonged survival for patients [10, 11] In particular, survival of patients after complete re-resection of tumor recurrence was comparable to patients not experiencing recurrences and was significantly improved compared to patients undergoing non-surgical treatment regimes for recurrence [12, 13] So far no serum biomarkers have been established for diagnosis and screening of TETs to identify those patients with higher risk for tumor recurrence. Consequently lifelong surveillance with repeated chest computed tomography (CT) with the inherent unwanted (carcinogenic) effects of radiation exposure is recommended for patients with TETs [5, 14, 15]. Sensitive and affordable molecular biomarkers for detection of TETs and their recurrences are needed [16].

CRP belongs to the pentraxin family and is produced by hepatocytes as part of the non-specific acute phase response. CRP production is triggered by proinflammatory cytokines, mainly interleukin (IL)-6, IL-1β and tumor necrosis factor alpha (TNFα) [17]. In daily routine, CRP is widely used as marker for inflammation, infection and tissue damage [18]. However, growing evidence indicates that CRP plays also a role in oncology. Indeed, the prognostic value of CRP has been already demonstrated for malignant pleural mesothelioma, lung, breast and pancreatic cancer for instance [19–22]. These results sparked our interest in the possible role of the proinflammatory molecule C-reactive protein (CRP) in the pathophysiology of TETs.

The present study was conducted in order to evaluate the value of CRP for estimating diagnosis, prognosis and surveillance of patients with TETs. This study also attempts to elucidate the possible source of CRP and the role of associated proinflammatory molecules in TETs.

## RESULTS

### Elevated CRP serum concentrations in patients with TETs compared to controls

**Figure 1:**
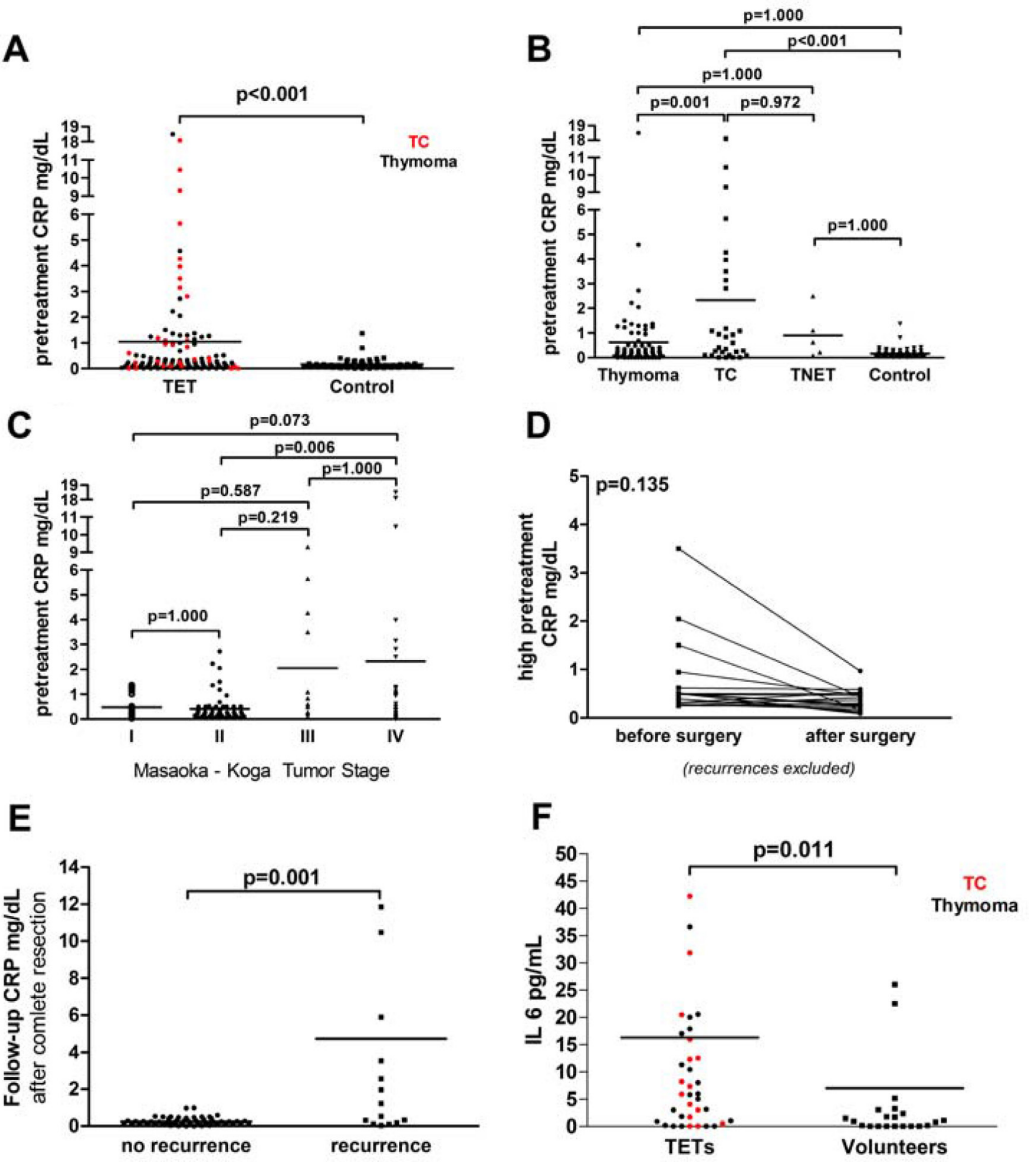
CRP serum concentrations in TETs CRP serum concentrations were significantly elevated in patients with TETs (*n* = 128) compared to controls (*n* = 64) (**A**). Separate analysis of thymomas (*n* = 93), TCs (*n* = 30) and TNETs (*n* = 5) compared to controls are shown (**B**). Highest CRP concentrations were found in metastatic TETs (Masaoka-Koga Stage IV; (**C**). In patients with high pretreatment CRP (≥ 0.22 mg/dL), CRP levels decreased after complete tumor resection (*n* = 52) (**D**), but increased significantly in case of tumor recurrence (*n* = 16) (**E**). IL-6 serum concentrations are significantly elevated in TETs (*n* = 39) compared to controls (**F**).

Pretreatment CRP serum concentrations were significantly elevated in patients with TETs (*n* = 128) compared to sex- and age-matched controls (*n* = 64) (TETs 1.03 ± 0.3 mg/dL vs. controls 0.16 ± 0.03 mg/dL; *p* < 0.001; Figure 1A). At one-way *ANOVA* CRP serum concentrations in patients with thymomas (*n* = 93; 0.62 ± 0.21 mg/dL), TCs (*n* = 30; 2.33 ± 0.7 mg/dL) and TNETs (*n* = 5; 0.90 ± 0.44 mg/dL) compared to controls were significantly different (*p* < 0.001*). Post hoc* comparisons revealed significant differences for TCs compared to thymomas (*p* = 0.001) and TCs compared to controls (*p* < 0.001; Figure 1B). Separate analysis of CRP serum concentrations of thymomas compared to controls and TNETs compared to controls by independent *student`s t-test* revealed significant differences (*p* = 0.010 and *p* = 0.016, respectively).

### Highest CRP serum concentrations in TCs and metastatic TETs

### WHO classification: different CRP serum concentrations

When compared with one-way *ANOVA* analysis, CRP serum concentrations were significantly different between WHO subtypes (*p* = 0.029), whereas *post hoc* comparisons showed no significance. We found the highest concentrations of pretreatment serum CRP in patients with TCs (2.33 ± 0.7 mg/dL) and the lowest CRP levels in WHO type AB and B2 thymomas (0.18 ± 0.03 mg/dL and 0.25 ± 0.08 mg/dL, respectively; Table 1). We detected significantly higher CRP serum concentrations in patients with B3 thymomas compared to patients with A/AB/B1/B2 thymomas (B3 thymomas vs. A/AB/B1/B2 thymomas: CRP 2.05 ± 1.14 mg/dL vs. 0.32 ± 0.049 mg/dL; *p* = 0.009). There was no statistically significant difference in preoperative serum concentrations of patients with B3 thymomas compared to patients suffering from TCs (B3 thymomas vs. TCs: CRP 2.05 ± 1.14 mg/dL vs. 2.33 ± 0.73 mg/dL; *p* = 0.825).

**Table 1:** Patient characteristics according to CRP serum concentrations

Characteristics	*n*	CRP (mg/dL) mean (median) ± SD (SEM)	*p*-value
**Age (years)**			
< 56	63	1.37 (0.24) ± 3.4 (0.43)	
≥ 56	65	0.71 (0.20) ± 1.5 (0.20)	0.165^a^
**Sex**			
Male	54	1.73 (0.18) ± 3.9 (0.58)	
Female	74	0.52 (0.19) ± 0.9 (0.11)	0.029^b^
**Tumor Subtype**			
MNT	6	0.66 (0.29) ± 0.74 (0.30)	
A	15	0.46 (0.19) ± 0.49 (0.13)	
AB	19	0.18 (0.15) ± 0.14 (0.03)	
B1	11	0.38 (0.18) ± 0.49 (0.15)	
B2	26	0.25 (0.10) ± 0.40 (0.08)	
B3	16	2.05 (0.34) ± 4.50 (1.14)	
TC	30	2.33 (0.72) ± 4.00 (0.73)	
TNET	5	0.90 (0.62) ± 0.98 (0.44)	0.029^c^
**Tumor Stage**			
I	27	0.57 (0.20) ± 0.92 (0.18)	
II	58	0.35 (0.17) ± 0.56 (0.07)	
III	13	2.01 (0.60) ± 2.87 (0.79)	
IV	30	2.33 (0.55) ± 4.79 (0.87)	0.003^c^
local (I–III)	98	0.63 (0.20) ± 1.32 (0.13)	
metastases (IV)	30	2.33 (0.55) ± 4.79 (0.87)	0.038^d^
non-invasive (I)	27	0.57 (0.20) ± 0.92 (0.18)	
invasive (II–IV)	101	1.15 (0.22) ± 2.95 (0.29)	0.317^a^
early stages (I–II)	85	0.42 (0.19) ± 0.70 (0.08)	
advanced stages (III–IV)	43	2.24 (0.60) ± 4.27 (0.65)	0.002^d^
**Myasthenia Gravis**			
Yes	34	0.91 (0.13) ± 3.3 (0.56)	
No	94	1.08 (0.27) ± 2.4 (0.25)	0.753^a^
**Tumor resection**			
before surgery	52	0.57 (0.20) ± 1.51 (0.21)	
after surgery *	52	0.26 (0.24) ± 0.22 (0.03)	0.135^e^
**Follow up (serum samples *n* =** 68)			
no recurrence	52	0.26 (0.24) ± 0.22 (0.03)	
recurrence	16	4.72 (1.60) ± 6.44 (1.61)	0.001^d^

Abbreviations: n, number; SD; standard deviation; SEM, standard error of the mean; MNT, micronodular thymoma; TC, thymic carcinoma; TNET, thymic neuroendocrine tumor; *recurrences were excluded; ^a^ independent-samples *T*-test; ^b^ Pearson's *χ^2^* test for independence; ^c^ one way *ANOVA*; ^d^ Whitney *U*-test; e paired-samples *T*-test.

Indeed, patients with the highest CRP serum concentrations (up to 18.5 mg/dL) had B3 or TC histology and were in Masaoka-Koga stages III and IV (Patient 1: male, 40.8 years, B3 thymoma, stage IV, recurrence at 137 months, alive, CRP 18.5 mg/dL; Patient 2: male, 52.6 years, TC, stage III, recurrence at 30 months, alive, CRP 9.30 mg/dL; Patient 3: male, 62.8 years, TC, stage IV, no recurrence, alive, CRP 10.45 mg/dL; Patient 4: male, 47.1 years, TC, stage IV, no recurrence, alive, CRP 18.1 mg/dL; Figure 1A, 1B).

### Masaoka-Koga stage: different CRP serum concentrations

CRP serum concentrations were significantly different according to clinical tumor stage (*p* = 0.003; Figure 1C; Table 1). Highest CRP serum concentrations were found in metastatic (stage IV) TETs compared to local (stage I-III) TETs (metastatic TETs 2.32 ± 0.9 mg/dL vs. local TETs 0.63 ± 0.13 mg/dL; *p* = 0.038). CRP serum concentrations were also elevated in advanced tumor stages (III-IV) compared to early tumor stages (I–II; *p* = 0.002). There was no significant difference between non-invasive (I) and invasive (II-IV) TETs (*p* = 0.317).

### Myasthenia gravis: no difference in preoperative CRP serum concentration

Of our patient cohort, 40 patients suffered from paraneoplastic MG and preoperative CRP values were available of 34 of them. Pretreatment CRP serum concentrations in patients with TETs with paraneoplastic MG (0.91 ± 0.56 mg/dL) were not significantly different compared to those patients without MG (1.08 ± 0.25md/dL; *p* = 0.753; Table 1). Pharmacological treatments specific for MG were as follows: corticosteroids (23.5%), azathioprine (20.5%) and acetylcholinesterase inhibitors (82.4%). Neither usage of corticosteroids (Yes vs. No: 0.24 ± 0.13 mg/dL vs. 1.11 ± 0.73 mg/dL; *p* = 0.517) nor azathioprine (Yes vs. No: 3.66 ± 2.59 mg/dL vs. 0.19 ± 0.06 mg/dL; *p* = 0.230) nor acetylcholinesterase inhibitors (Yes vs. No: 1.08 ± 0.68 mg/dL vs. 0.10 ± 0.07 mg/dL; *p* = 0.514) had a significant impact on pre-operative CRP serum concentrations.

### CRP serum concentrations are elevated in patients with recurrence

In a subgroup of 68 patients, pre - and postoperative CRP concentrations were available (median follow up of 14.8 months). Recurrences occurred in 16 patients (local: *n* = 2, regional: *n* = 4, distant: *n* = 10), whereas 52 patients showed no signs of tumor recurrence. In these 52 patients without recurrence, CRP serum concentrations decreased from 0.57 ± 0.21 mg/dL preoperatively to 0.26 ± 0.03 mg/dL postoperatively (*p* = 0.135; Figure 1D). In case of tumor recurrence, postoperative CRP serum concentrations increased significantly (4.72 ± 1.61 mg/dL; *p* = 0.001; Table 3; Figure 1E). Patients with distant tumor recurrence showed higher but not significantly different CRP concentrations compared to those with regional and local recurrences (5.7 ± 2.3 mg/dL compared to 3.12 ± 2.9 mg/dL and 3.05 ± 0.5 mg/dL, respectively; *p* = 0.764). In our patient cohort 2 out of 16 patients had distant recurrences with liver metastases (CRP 10.3 ± 9.8 mg/dL), which was not associated with higher levels of CRP (*p* = 0.571).

**Table 3:** Univariable and multivariable Cox regression analyses

	Univariable Model				Multivariable Model			
			95% CI				95% CI	
	HR	*p*^a^	Lower	Upper	HR	*p*^a^	Lower	Upper
**Overall Survival**								
Sex (Male)	1.134	0.822	0.380	3.377	0.709	0.647	0.103	4.899
Age (continuous)	1.038	0.080	0.996	1.081	1.062	0.023	0.992	1.136
Myasthenia Gravis (No)	6.361	0.077	0.820	49.340	2.531	0.406	0.143	44.921
Histology (Thymoma vs. TC)	0.183	0.003	0.059	0.568	0.246	0.009	0.028	2.138
Resection Status (R0 vs. R1-2)	0.194	0.008	0.058	0.653	0.566	0.518	0.059	5.464
Tumor Stage (I + II vs. III–IV)	0.380	0.090	0.124	1.165	0.406	0.250	0.054	3.062
Tumor Size (continuous)	1.010	0.368	0.988	1.033	1.019	0.241	0.977	1.063
CRP (low vs. high)^b^	0.477	0.240	0.139	1.639	0.380	0.180	0.059	2.436
**Cause Specific Survival**								
Sex (Male)	1.732	0.472	0.387	7.742	1.272	0.869	0.072	22.347
Age (continuous)	1.036	0.207	0.980	1.096	1.112	0.045	1.002	1.233
Myasthenia Gravis (No)	2.843	0.334	0.342	23.651	0.109	0.282	0.002	6.208
Histology (Thymoma vs. TC)	0.067	0.001	0.013	0.352	0.011	0.031	0.000	0.664
Resection Status (R0 vs. R1-2)	0.071	0.001	0.016	0.319	0.013	0.044	0.000	0.897
Tumor Stage (I + II vs. III–IV)	0.098	0.032	0.012	0.820	0.224	0.357	0.009	5.422
Tumor Size (continuous)	1.002	0.923	0.969	1.035	1.058	0.946	0.205	5.453
CRP (low vs. high)^b^	0.561	0.530	0.093	3.397	0.759	0.867	0.030	19.392
**Freedom From Recurrence**								
Sex (Male)	1.684	0.192	0.770	3.683	0.984	0.980	0.281	3.449
Age (continuous)	0.964	0.008	0.938	0.991	0.947	0.012	0.908	0.988
Myasthenia Gravis (No)	2.127	0.127	0.806	5.611	3.362	0.215	0.495	22.826
Histology (Thymoma vs. TC)	0.275	0.003	0.116	0.651	0.427	0.140	0.138	1.324
Resection Status (R0 vs. R1-2)	0.361	0.048	0.131	0.992	0.477	0.302	0.117	1.944
Tumor Stage (I + II vs. III–IV)	0.307	0.004	0.136	0.689	0.739	0.588	0.242	2.201
Tumor Size (continuous)	1.005	0.456	0.992	1.017	1.013	0.162	0.995	1.032
CRP (low vs. high)^b^	0.303	0.015	0.116	0.792	1.313	0.710	0.311	5.538

Abbreviations: HR, hazard ratio; *p, p-*value; CI, confidence interval; ^a^ Cox-Regression; ^b^ median CRP (0.22 mg/dL) was used for grouping patients into high and low CRP cohorts.

### Diagnostic accuracy of CRP to predict tumor recurrence

Based on these results, we next evaluated the sensitivity, specificity, and positive as well as negative predictive value (PPV and NPV) of elevated CRP serum levels to predict tumor recurrence. At a cutoff level of CRP of ≥ 0.5 mg/dL, the sensitivity was 62.5% (10 out of 16), the specificity was 92.4% (48 out of 52), the PPV was 71.4% (10 out of 14) and the NPV was 88.9% (48 out of 54).

### Prognostic characteristics in patients with TETs

### Survival analysis

**Figure 2:**
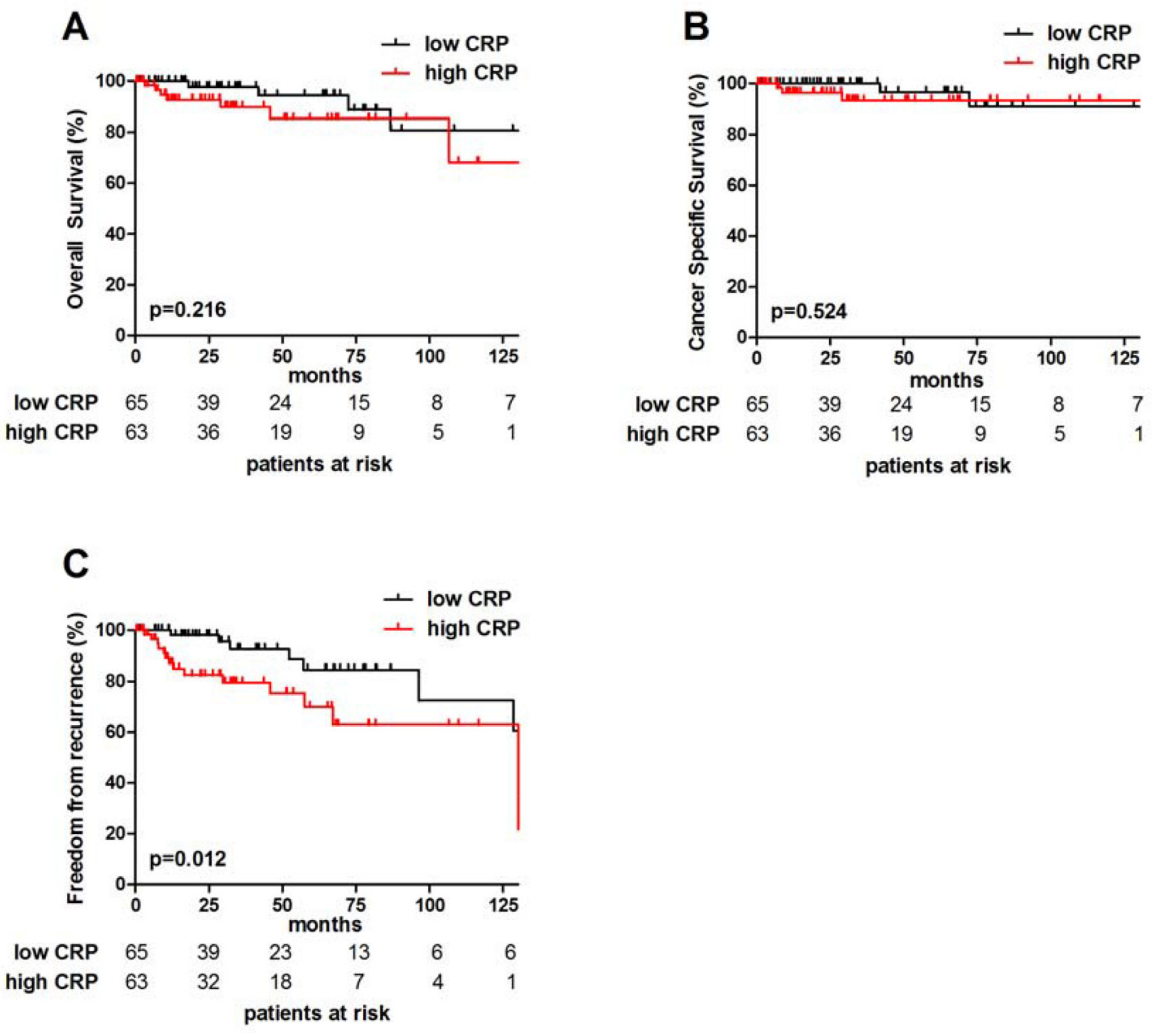
Prognostic impact of CRP in TETs Median CRP (0.22 mg/dL) was used for grouping patients into low and high CRP cohorts. Overall Survival, Cause Specific Survival and Freedom From Recurrence are shown (**A**–**C**). Log-rank test was performed for analyses.

Histologic tumor subtype (Thymoma vs. TC) and completeness of surgical tumor resection were significant factors for OS (*p* = 0.001 and *p* = 0.003), cause-specific survival (CSS; *p* < 0.001 and *p* < 0.001) and FFR (*p* = 0.002 and *p* = 0.039), respectively. Advanced Masaoka-Koga tumor stage (III+IV) was associated with significantly worse CSS (*p* = 0.008) and FFR (0.002). Pretreatment CRP had no influence on OS and CSS in patients with TETs (*p* = 0.201 and *p* = 0.501, respectively). For prognostic and survival analysis, the median pretreatment CRP value of 0.22 mg/dL was used to dichotomize patients into high and low CRP groups. Accordingly, 5- and 10- year FFR in TETs with high pretreatment CRP was significantly worse compared to TETs with low CRP, which was as follows: 84.5% and 72.4% compared to 68.1% and 68.1% (*p* = 0.010; Figure 2; Table 2).

**Table 2:** Univariable survival analyses

	Overall Survival			Cause Specific Survival			Freedom From Recurrence		
	5 year	10 year	*p*^a^	5 year	10 year	*p*^a^	5 year	10 year	*p*^a^
**Histology**									
TC	66.5	49.9		69.5	52.2		63.3	63.3	
Thymoma	94.2	82.9	0.001	95.9	95.9	0.001	87.5	79.4	0.001
**Residual status**									
R0	92.8	81.7		97.4	94.4		81.7	74.7	
R1 + 2	68.1	45.4	0.003	68.1	45.4	0.001	46.8	46.8	0.039
**Tumor Stage**									
I–II	92.5	84.0		97.6	97.6		87.5	83.7	
III–IV	86.4	68.9	0.079	88.4	77.5	0.008	62.5	52.1	0.002
**CRP** *									
high	85.8	68.6		93.5	93.5		68.1	68.1	
low	94.5	80.8	0.201	96.6	90.9	0.501	84.5	72.4	0.010

Abbreviations: *p, p-*value; ^a^ Log-rank test; TC, thymic carcinoma; *median CRP (0.22 mg/dL) was used for grouping patients into high and low CRP cohorts.

There was no significant difference between OS and CSS in patients who received adjuvant therapy compared to those patients who did not (*p* = 0.424 and *p* = 0.117; respectively). Conversely, median FFR was significantly worse in patients who received adjuvant therapy (128.5 months; 95% CI 40.7–216.3 months) compared to median FFR of 160.7 months (95% CI 30.6–290.8 months) in patients who did not receive adjuvant therapy (*p* = 0.001).

### Prognostic characteristics

Univariable and multivariable analysis of sex (male vs. female), age (continuous), MG (pos. vs. neg.), tumor histology (TC vs. Thymoma), completeness of resection (R0 vs. R1+2), Masaoka-Koga tumor stage (I+II vs. III–IV), tumor size (continuous) and pretreatment CRP (low vs. high) for OS, CSS and FFR are shown in Table 3.

At univariable analysis, presence of TC and incomplete resection were significant prognostic factors for worse OS (*p* = 0.003 and *p* = 0.008; respectively), CSS (*p* = 0.001 and *p* = 0.001; respectively) and FFR (*p* = 0.003 and *p* = 0.048; respectively). Advanced Masaoka-Koga tumors stage (III+IV) was also associated with shorter CSS (*p* = 0.032) and FFR (*p* = 0.004). High pretreatment CRP serum concentrations were prognostic with regard to FFR (*p* = 0.015), while OS and CSS were not affected.

Multivariable analysis demonstrated that presence of TC was still significantly predicting worse OS and CSS (*p* = 0.009 and *p* = 0.031; respectively), while there was no effect on FFR. Incomplete tumor resection was associated with significantly worse CSS (*p* = 0.044), while OS and FFR was not affected. CRP serum concentrations were not an independent prognostic marker at multivariable analysis. Presence of paraneoplastic MG, sex or tumor size did not effect OS, CSS or FFR at univariable or multivariable analysis. Age as continuous variable significantly influenced OS, CSS and FFR at multivariable analysis (*p* = 0.023; *p* = 0.045 and *p* = 0.012, respectively; Table 3).

### Elevated IL-6 serum concentrations in patients with TETs did not correlate with CRP

To evaluate the possible association of increased CRP serum concentrations with proinflammatory cytokines we measured IL-6 and IL-1β serum concentrations in 39 patients with TETs (thymoma: *n* = 23; TC: *n* = 15) in comparison to age- and sex-matched volunteers. Interestingly, IL-6 serum concentrations were significantly elevated in patients with TETs compared to volunteers (16.27 ± 6.1pg/mL vs. 7.1 ± 4.0 pg/mL; *p* = 0.011, Figure 1F), while we did not detect any differences for IL-1β serum concentrations (*p* = 0.237). IL-6 and IL-1β serum concentrations did not correlate with pretreatment CRP serum concentrations (*p* = 0.724; *r* = 0.062 and *p* = 0.586; *r* = 0.080, respectively).

### TETs do not express CRP

**Figure 3:**
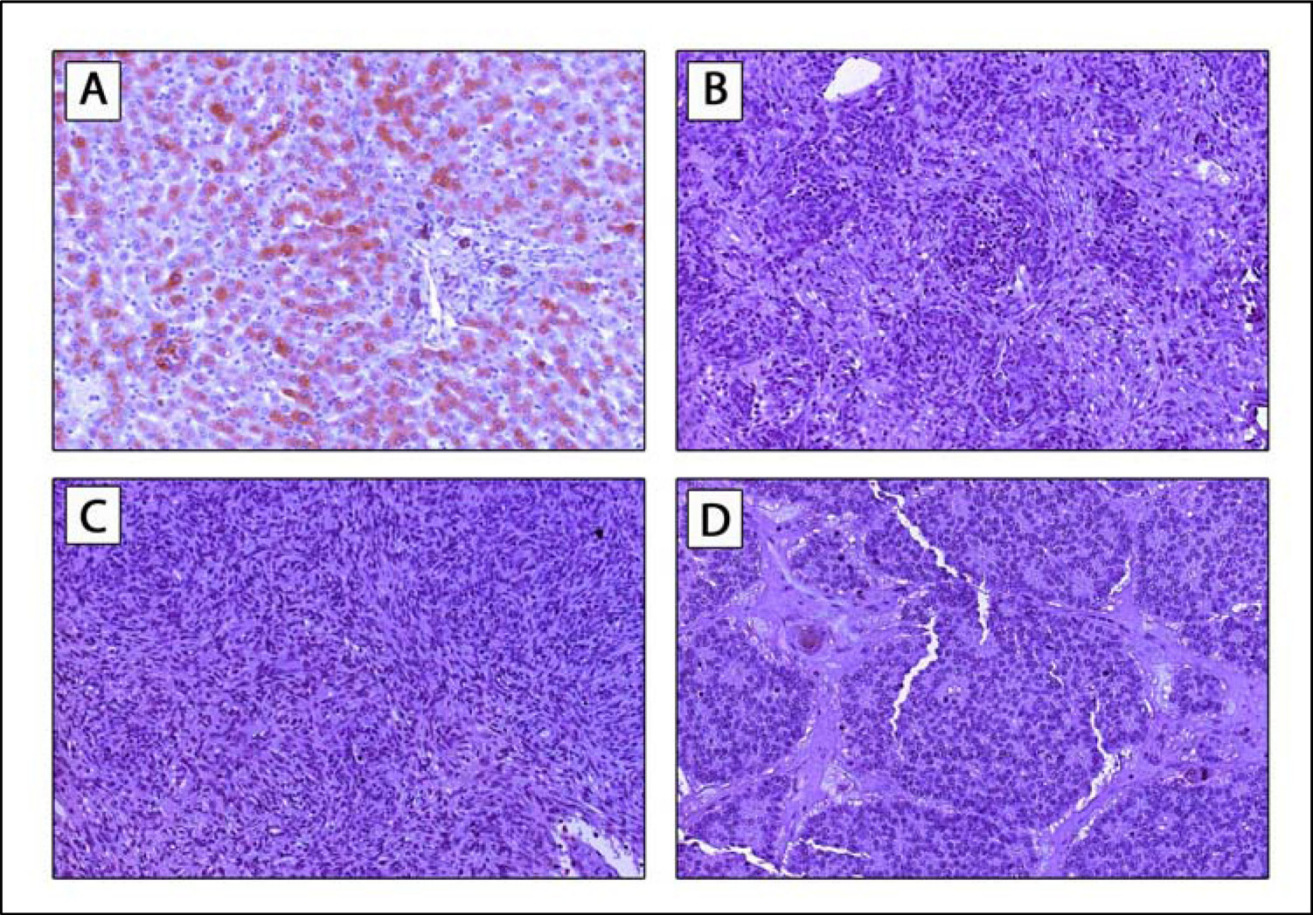
CRP expression in TETs CRP staining was absent in all TETs. Positive staining was only found in physiologic liver specimens (**A**; 200× magnification), which were used as positive control. Staining of WHO type AB thymoma (**B**), type B2 thymoma (**C**) and TC (**D**) are shown (all 200× magnification). *TETs*, thymic epithelial tumors; *TC*, thymic carcinoma.

Finally we performed CRP staining of a subset of 33 thymic specimens (Thymoma: *n* = 27; TC: *n* = 6) to assess whether TETs express CRP. CRP staining was missing in all cases of TETs and CRP expression was only found in liver specimens that were used as positive controls (Figure 3).

## DISCUSSION

Our results are in line with previous reports on CRP in cancer, where high CRP serum concentrations were associated with more aggressive tumor behavior, higher tumor stages and poor outcome. In patients with malignant pleural mesothelioma, pancreatic cancer, non-small cell lung cancer and hepatocellular carcinoma elevated CRP levels were associated with significantly worse prognosis [19, 20, 22, 25] Multivariable analysis revealed CRP as an independent prognostic predictor in patients with malignant diseases of much poorer overall prognosis than TETs: malignant pleural mesothelioma (CRP ≥ 1 mg/dL; OS) [19]; non-small cell lung cancer (CRP > 1 mg/dL; CSS) [20]; adenocarcinoma of the pancreas (CRP > 4.5 mg/dL; CSS) [22]; hepatocellular carcinoma (CRP > 0.2 mg/dL; FFR). [25] Altogether, elevated preoperative CRP levels was associated with higher mortality in patients with malignancies and CRP was also a useful marker to identify tumor recurrences [26].

This study also focuses on preoperative CRP values. The reasoning for investigating preoperative CRP values lies in the early postoperative rise after surgery and that the multiple surgical methods currently in use (e.g. minimally-invasive vs. open thoracic surgery) impose different degrees of surgical stress on the patients. Surgical stress that results quantitatively in different acute phase responses and CRP values [27].

In TETs, thymoma histotype was suggested to be of prognostic value [28]. While CRP serum concentrations could not differentiate between TETs with poorer prognosis (B3 and TCs), thymoma types with better prognosis: A/AB/B1/B2 could be differentiated from those with poorer prognosis: B3 and TCs [14, 28].

Tumor recurrence is a characteristic feature of TETs and occurs in 10–30% of cases even decades after primary resection [3, 10] Surgical resection of tumor recurrence provides an excellent therapeutic option, which is effective, safe and leads to good long time OS [12, 29]. In patients with thymomas excellent 5- and 10-year OS of 88–92.8% and 75–90.5% was reported after re-resection of tumor recurrence [3, 14]. Hence reoperation of tumor recurrence provides an excellent therapeutic option and clearly underlines the necessity of appropriate markers to detect recurrences at early tumor stages. Our data warrant further study to evaluate the use of CRP serum concentrations for oncologic follow-up of patients after treatment of TETs. CRP measurements can be readily included into structured follow-up routines. Due to the fact that CRP is an unspecific marker, it is necessary that clinicians rule out other reasons for increased serum CRP before thinking of cancer or tumor recurrence. Therefore, the information of CRP serum concentrations in oncologic patients can only be interpreted in relation to tumor stage or prognosis after all the information of possible other reasons for CRP elevation has been valued. Nevertheless, if in a patient with elevated CRP an infection can be ruled out a high suspicion for recurrence will remain that will prompt the clinical and radiological examinations to diagnose or rule out tumor recurrence.

However, we found accurate PPV of 71.4% and NPV of 88.9%, indicating that CRP could be a cheap, commonly available and established routine serum marker that might be helpful, especially in oncologic follow-up to identify patients with increased risk of tumor recurrence.

Chest X-rays and chest CT scans are currently the only recommended tool for oncologic follow-up in patients with TETs [23]. Today no adequate markers exist that have changed clinical tertiary prevention or the use of chest CT scans. We believe that prospective trials for tertiary prevention are warranted in patients with increased CRP serum concentrations at diagnosis of primary disease. Such a protocol would control serum CRP values at regular intervals and may probably omit CT scans. It certainly would not completely replace surveillance by CT. According to data of the European Society of Thoracic Surgeons (ESTS) Thymic Working Group on prognostic factors in thymic malignancies in more than 2000 cases, the majority of institutions performed CT scans 3- to 6-months after tumor resection for the first 3 years, followed by lifelong annual CT scans [14]. Similarly, the International Thymic Malignancies Interest Group (ITMIG) recommends a minimum of annual chest CT scans for 5 years after tumor resection followed by alternating chest CT scans with annual chest radiographs until the 11th postoperative year. After that annual chest radiographs alone are recommended. Resected stage III or IVa thymoma, thymic carcinoma, incomplete resection, or other high-risk tumors are suggested to undergo additional CT imaging every 6 months for 3 years [23]. Based on recommendations of ESTS [14] and ITMIG [23], we propose the following protocol to evaluate the role of CRP for oncologic follow-up: Chest CT scans every 6 months for the first 3 years followed by alternating chest CT scans with chest radiographs until the 11th postoperative year followed by annual chest radiographs only. Each check up should be accompanied by a blood draw to detect CRP serum concentrations.

We suggest that CRP serum levels should be further determined every 6 months. Elevated CRP serum concentrations in the absence of other reasons for increased inflammatory parameters may indicate tumor recurrence. We believe that CRP measurements will increase the chance of detecting recurrences earlier especially when radiological sensitivity is low, as is the case in surveillance with chest x-rays. Earlier detection of tumor recurrence may be associated with better treatment options and improved outcome for patients.

Moreover we attempted to elucidate the pathogenesis and origin of “tumor-CRP” in patients with TETs. We did not detect CRP expression in TETs, although CRP expression has been already demonstrated in other solid tumors, including hepatocellular carcinoma, esophageal cancer or renal cell cancer [30–32]. Conversely, we detected significantly elevated IL-6 serum concentrations in patients with TETs, while IL-1β levels were not significantly different. Surprisingly and in contrast to diverse reports, which already demonstrated a positive correlation between IL-6 and CRP serum levels in patients with breast cancer or lung cancer, we did not detect significant correlations between IL-6 and CRP [33, 34] Two case reports of patients with TETs (TC and TNET) reported active secretion of IL-6 by neoplastic thymic epithelial cells. [35, 36] CRP levels, white blood cells and IL-6 levels were increased in the patient with TC and decreased significantly after tumor resection [35]. Overproduction of IL-6 has been also linked to the pathogenesis of MG. Indeed, surgically induced overproduction of IL-6, accompanied by increase of CRP was proposed to prompt myasthenic crisis [37].

Altogether the origin and pathogenesis of “tumor CRP” in patients with TETs remain elusive. Whether increased CRP serum concentrations result from active secretion of tumor cells or activated peripheral blood mononuclear cells (PBMCs) or whether increased CRP levels result from tumor growth, progression and associated immune response is unclear [38–41] In accordance with our data, we speculate that elevated CRP levels in TETs most likely results from cancer-related inflammation, caused by the interaction of immune cells (e.g. PBMCs) and tumor cells, which in turn leads to increased CRP production by hepatocytes. Our previous works are also in line with the cancer-related inflammation thesis in TETs. The search for biomarkers in TETs is work in progress. While existing publications [44–46] have focused on genetic analyses on tumor tissue we sought to search for biomarkers detectable in serum of patients with TETs [16, 24]. To our knowledge there is no widespread use of serum biomarkers in clinical routine. We could show that proinflammatory HSPs and RAGE-ligands [42, 43] were significantly elevated in patients with TETs and results were again pronounced in advanced tumor stages and more aggressive tumor subtypes [16, 24].

Our study carries of course the weaknesses of retrospective studies and single center experiences on orphan diseases, such as the inherent bias of selection and information. However, the strength of the present study is that for the first time the prognostic and diagnostic value of CRP has been described for patients with TETs. Prospective multicenter studies are warranted to assess CRP concentrations in larger patient cohorts and to define common definitions and cut-offs for increased CRP concentrations.

To conclude, we found highest pretreatment CRP serum concentrations in patients with metastatic tumors and TCs. High pretreatment CRP was associated with significantly worse FFR and CRP serum concentrations increased significantly in case of tumor recurrence. The use of CRP in the diagnosis and oncologic follow-up of patients with TETs is promising and warrants future study.

## MATERIALS AND METHODS

### Study population

We included 149 patients who underwent surgical tumor resection at the department of thoracic surgery (Medical University Vienna) between June 1990 and May 2015 (90% were treated 2002–2015) with median follow-up time of 35 months (range 1–249 months). Eighty patients (53.7%) received multimodality treatment, consisting of surgery with chemotherapy and/or radiotherapy according to neoadjuvant or adjuvant therapy regimens. Surgical tumor resection alone was performed in 46.3% (*n* = 69) of patients. Patient demographics are depicted in Table 4. Chest CT scans were performed in each patient for preoperative staging. In selected cases, when advanced tumor stages were clinically suspected, positron emission tomography (PET) CT or at least abdominal and head CT scans were additionally performed for staging. Within oncologic follow up, all patients received periodically chest CT-scans. All patients with verified TETs or mediastinal tumors suspected to be TETs were preoperatively seen by a neurologist for the diagnosis or exclusion of MG.

**Table 4:** Characteristics of the patient cohort (*n* = 149)

Characteristics	Numbers	%
**Age**, years (mean ± SD)	56 ± 15	–
**Sex**		
Male	65	43.6
Female	84	56.4
**Tumor Subtype**		
MNT	6	4.0
A	18	12.1
AB	21	14.1
B1	12	8.1
B2	32	21.5
B3	19	12.8
TC	35	23.5
TNET	6	4.0
**Masaoka- Koga Tumor Stage**		
I	31	21.5
II	61	40.9
III	21	14.1
IV	36	23.5
**Therapy**		
Surgery only	69	46.3
Multimodality Treatment	80	53.7
Tumor Size, mm (mean ± SD)	60.8 ± 30.9	-
**Residual Tumor Classification**		
R0	131	87.9
R1	15	10.1
R2	2	1.3
Only Biopsy	1	0.7
**Myasthenia Gravis**		
Yes	40	26.8
No	109	73.2
**Recurrence**		
Yes	27	18.1
No	122	81.9
**Type of Recurrence**		
Local	7	25.9
Regional	10	37.0
Distant	10	37.0

Abbreviations: SD, standard deviation; MNT, micronodular thymoma; TC, thymic carcinoma; TNET, thymic neuroendocrine tumor. Tumor Size, the largest tumor diameter (mm) on preoperative chest CT-scans.

### Outcome analysis

We followed the definitions of recurrence and outcome as recommended by the ITMIG [23]. To assess the prognostic value of CRP we analyzed OS, CSS and FFR. OS as the primary outcome was calculated from date of surgery to date of death of any cause. The endpoint of interest for CSS was defined as death from TET (censored observations: unrelated deaths and unknown cause of death). [23]. FFR was calculated only in patients after complete surgical resection (R0) from date of surgery to date of recurrence and full information on recurrence status [14].

### CRP serum concentrations and exclusion criteria of the study

CRP serum concentrations were measured at the institutional department of laboratory medicine using a latex-enhanced immunoturbidimetric assay (Roche, Mannheim, Germany) according to the manufacturer's instructions. CRP serum concentrations are influenced by a broad spectrum of diseases and interventions. There had to be at least 4 weeks between last applications of ChT or surgery and the blood draw to generate serum. Clinical conditions that led to exclusion from the study: pneumonia, COPD exacerbation and acute cardiac insufficiency. Patients with acute infections (4 pneumonia, 3 urinary tract infection) based on medical reports were excluded from CRP analysis. In total 149 patients underwent surgical tumor resection at our department between 1990 and 2015. Preoperative CRP serum concentrations were missing in 14 patients with TETs. Hence, 21 patients were excluded and preoperative serum CRP concentrations were available from 128 patients with TETs. Postoperative (=follow-up) serum samples have been collected since 2012. Hence pre- and postoperative CRP serum concentrations were only available from a subset of 68 patients (16 recurrence; 52 no recurrence) with median follow-up of 14.8 months [range 0.13–105.6 months]. CRP serum concentrations of 64 sex- and age matched healthy volunteers served as controls.

### Immunohistochemistry

Immunohistochemical staining was performed according to standard protocols [16, 24] Briefly, 3-μm thick formalin-fixed and paraffin embedded tissue specimens of TETs were deparaffinized. Antigen retrieval was performed by using citrate buffer (pH 6.0, Target Retrieval Solution, DAKO, Glostrup, Denmark) and by boiling slides at 600 watt (3 × 5 min) in microwave oven. Endogenous peroxidase was quenched by applying 0.3% hydrogen peroxide. Sections were incubated with monoclonal rabbit anti-human CRP antibody (Clone Y284, Abcam, Cambridge, UK) for 1 h at room temperature and goat anti-rabbit antibody was used as secondary antibody (Vectastatin ABC kit, Vector Laboratories, Burlingame, USA). Immunoreactivity was amplified by using biotin-avidin peroxidase conjugates (Vectastatin ABC kit, Vector Laboratories, Burlingame, USA) and 3,3´-diaminobenzidine (DAB) was used as chromogen (DAB Peroxidase substrate kit, Vector Laboratories, Burlingame USA). Sections were finally counterstained with hematoxylin, rehydrated and mounted. Liver specimens were used as positive control.

The monoclonal rabbit anti-human CRP antibody (Clone Y284, Abcam, Cambridge, UK) was already used to analyze CRP expression in hepatocellular carcinomas [30, 47].

### ELISA

Enzyme linked immunosorbent analysis (ELISA) was performed to assess preoperative IL-6 and IL-1β serum concentrations in 39 patients with TETs compared to healthy controls. Commercially available human IL-6 (R&D Systems, Minneapolis, MN, USA, S6050) and human IL-1β ELISA kits (R&D Systems, Duo Set ELISA, DY201) were used. All tests were performed according to manufacturers’ instructions and all samples were measured in duplicates.

### Statistical methods

Statistical analysis of data was performed by using SPSS software (version 21; IBM SPSS Inc., IL, USA). *Whitney U-*Test and *Kruskal-Wallis rank test* were used to evaluate non-normal distributed variables between two or more than two groups. Unpaired *student's t* test and one-way *ANOVA* were used to compare means of two or more than two independent groups with normal distribution. *Post hoc* comparisons were computed with Tukey’s-B and Bonferroni correction. All data were reported as mean ± standard error of the mean (SEM) in the text. *Chi-square test* was used to compare nominal variables. *Pearson correlation* (correlation coefficient, r) was performed to analysis linear relationships between two numerical variables. *Kaplan-Meier survival analysis* and *Log-rank test* were used to analyze OS, CSS and FFR. *Cox regression* was performed to assess the prognostic impact of different factors on outcome and to calculate *Hazard Ratios* (HR) with corresponding 95% confidence intervals (CI). *Univariable* and *multivariable analyses* were calculated for the following parameters: sex (male vs. female), age (continuous), Myasthenia Gravis (positive vs. negative), histology (TC vs. Thymoma), resection status (R0 vs. R1 + 2), tumor stage (I–II vs. III–IV), tumor size (continuous) and pretreatment CRP (low vs. high CRP). The median pretreatment CRP value of 0.22 mg/dL was used to dichotomize patients into high and low CRP groups. Conversely, the institutional cut-off value of 0.5 mg/dL was used to test the diagnostic accuracy (*sensitivity, specificity, PPV* and *NPV*) of CRP. All tests were two-sided and *p-value*s below 0.05 were considered as statistically significant.
